# Umbilical Cord Prolapse in a Labouring Patient: A Multidisciplinary and Interprofessional Simulation Scenario

**DOI:** 10.7759/cureus.1692

**Published:** 2017-09-16

**Authors:** Chandrew Rajakumar, Adam Garber, Purnima M Rao, Genevieve Rousseau, George A Dumitrascu, Glenn D Posner

**Affiliations:** 1 Department of Obstetrics & Gynecology, University of Calgary; 2 Department of Obstetrics & Gynecology, The Ottawa Hospital / University of Ottawa; 3 Department of Anesthesiology and Pain Medicine, The Ottawa Hospital / University of Ottawa; 4 Department of Innovation in Medical Education, University of Ottawa

**Keywords:** postgraduate medical education, simulation scenario, obstetrics and gynecology, anesthesiology, nursing, crisis resource management

## Abstract

This case is one of an eight-case multidisciplinary curriculum designed and implemented at the University of Ottawa by simulation educators with specialty training in obstetrics and gynecology (OB/GYN) and anesthesiology. Consultation from a nurse educator maintained quality and relevance of objectives for nursing participants.

The curriculum was prepared to train OB/GYN and anesthesiology residents and nurses to hone crisis resource management skills and to recognize and manage rare/critical medical events in an obstetrical setting. Obstetricians, anesthesiologists, and nurses often work together in acute, high-stakes situations, and this curriculum provides a safe environment to practice team-based management of such emergencies.

Over an eight-year period, this curriculum has been executed in scenario couplets on a four-year cycle to allow OB/GYN and anesthesiology residents exposure to all scenarios during a five-year residency beginning in their second year. Prospective evaluative data has been positive. For example, over 90% of participants rated these simulations to be 5 out of 5 with comments, such as “Was an effective use of my educational time” and “Will influence/enhance my future practice”.

In this scenario, participants must recognize and manage fetal distress resulting from umbilical cord prolapse in a labouring patient and respond with urgent operative delivery. This scenario requires adult and fetal mannequins with presenting umbilical cord for pelvic examination as well as equipment for fetal monitoring, general anesthetic, and emergency cesarean section.

This simulation case includes a case template, critical actions checklist, debriefing guide, summary of key medical content, and an evaluation form for learners to provide feedback.

## Introduction

Umbilical cord prolapse can be occult or overt and occurs when a segment of umbilical cord advances alongside or ahead of the presenting part of the fetus. This makes it susceptible to compression by the presenting part and may result in fetal asphyxia. This event occurs in 0.18% of live birth deliveries [1]. Though rare, the sequelae can be significant and perinatal mortality has been reported as high as 3% [2].

Due to the rarity of this event and the significance of its outcomes, it is paramount that nurses and trainees in obstetrical care be prepared to effectively identify and manage this scenario. In the event of fetal asphyxia warranting expedited delivery, anesthesiology trainees should be exercised in the provision of rapid anesthesia to facilitate safe delivery. This includes understanding and adapting to the physiologic changes of pregnancy.

This scenario is part of a four-year interdisciplinary curriculum for obstetrics/gynecology (OB/GYN) and anesthesiology residents developed at the University of Ottawa Skills and Simulation Centre (see Table 1). Obstetricians and anesthesiologists often work together in acute, high stakes situations. This curriculum was designed in order to allow residents in both fields to practice both technical and crisis resource management skills in a safe and risk-free environment. Further, the interdisciplinary aspect of this curriculum allows both fields to learn about each other's knowledge, roles, and priorities during a crisis.

**Table 1:** OB/GYN Anesthesiology Nursing Simulation Curriculum MgSO4: magnesium sulfate; OB/GYN: obstetrics/gynecology

YEAR	TOPIC 1	TOPIC 2
YEAR 1	Autonomic Dysreflexia	Twin Breech Delivery
YEAR 2	MgSO4 Toxicity	Difficult Airway, Emergent Delivery
YEAR 3	Thyroid Storm	Amniotic Fluid Embolism
YEAR 4	Cord Prolapse with Abnormal Fetal Heart Rate	Postpartum Hemorrhage

The target audience is OB/GYN residents, anesthesiology residents, and practicing nurses as part of a comprehensive interdisciplinary curriculum of theatre-based simulation. Three to five learners participate in each session, and the duration of training is one hour.

The goals of the scenario are for the team to recognize and appropriately manage a prolapsed umbilical cord in a labouring patient and to demonstrate effective crisis resource management skills in a situation wherein human resources are taxed.

Team objectives

(Adapted from the Ottawa Global Rating Scale [3])

1. Demonstrates leadership skills by remaining calm and in control, making firm decisions, and maintaining a global perspective.

2. Demonstrates problem-solving skills by identifying the prolapsed fetal umbilical cord by conducting a targeted physical examination and/or evaluation of abnormal fetal heart rate pattern and considering most likely alternatives in crisis.

3. Demonstrates situational awareness skills by avoiding fixation error, reassessing and reevaluating situations, and anticipating likely events.

4. Demonstrates resource utilization skills by using human and physical resources to maximal effectiveness, setting clear task priority, and asking for help early.

5. Demonstrates communication skills by communicating clearly and concisely, encouraging input and listening to staff feedback, and using directed verbal/non-verbal communication.

OB/GYN resident objectives

1. Demonstrate the management of intrapartum umbilical cord prolapse.

2. Communicate effectively the roles and/or transfer of leadership to team members while applying pressure on the fetal head.

3. Recognize the need for an emergent cesarean section.

Anesthesiology resident objectives

1. Demonstrate the ability to independently provide anesthesia care for obstetrical patients during a crisis.

2. Demonstrate knowledge of how to develop and execute a plan for general endotracheal anesthesia based on the physiology and physical changes of pregnancy.

3. Communicate concerns with multidisciplinary team members effectively.

Nursing objectives

1. Communicate effectively with team members during an obstetrical emergency.

2. Manage multiple nursing interventions simultaneously.

3. Demonstrate seeking assistance appropriately and in a timely manner.

4. Demonstrate how to assist in an emergency cesarean section performed under general anesthesia.

## Technical report

Case summary

A 33-year-old gravida 2 para 1 patient is in spontaneous and active labour at 39-weeks’ gestation. She has declined epidural and her birth-plan is to experience natural childbirth. Prenatal testing indicates she is group-B streptococcus (GBS)-positive and therefore she has an intravenous (IV) catheter for penicillin G administration. Pregnancy and labour have been unremarkable. The scenario begins with an abrupt reduction in the fetal heart rate from the baseline. This prompts the patient’s nurse to initiate her usual reaction and ultimately contact the OB/GYN resident on-call. Nursing or OB/GYN physical examination should identify the fetal head as well as a segment of umbilical cord protruding from the cervix. Usual maneuvers to alleviate cord compression should be performed and the team must realize the need for an emergency cesarean section. The charge nurse, “attending” OB/GYN, and anesthesiologist should be notified. The plan should be communicated to the patient and she must be willing to proceed. Once in the operating room, the anesthesiologist will perform a history and administer a general anesthetic. Following this, the obstetrical team will perform an emergency cesarean section while one team member continues to apply pressure on the fetal head.

Learner preparation

We advocate for a comprehensive pre-briefing prior to any theatre-based simulation session, as described by Rudolph, et al. [4].

Nurse: You are working in a delivery room. Your patient, Mrs. Osgood, is a 33-year-old G2P1 patient at 39 weeks’ gestation. She has had an uncomplicated pregnancy with usual antenatal care. She is admitted with “spontaneous rupture of membranes”, leaking clear fluid and in active labour. She has tested GBS positive and has received one dose of penicillin G. She was 5 cm dilated at last examination (approximately one hour ago).

Junior OB/GYN resident: You are the senior OB/GYN resident on call in a tertiary hospital. Your staff OB/GYN is also in the hospital.

Senior OB/GYN resident: You are the OB/GYN attending physician on call in a tertiary hospital. You have a senior OB/GYN resident also on call with you.

Anesthesiology resident: You are the attending anesthesiologist on call for OB/GYN in a tertiary hospital.

Setup

Equipment/Environment

This scenario takes place during a call shift in a tertiary care hospital obstetrical labour room and operating room.

Two rooms are required (#1 setup as a labour room, #2 setup as an operating room)

Operating Room

A single simulation room (initially set up as a labour room with hidden operating room equipment in the periphery that will be uncovered and moved into place once the team “exits” the labour room)

Part 1 – High fidelity mannequin (wig, pregnant abdomen with vertex baby inside and cord presenting) on the labour bed. Awake, breathing spontaneously with standard monitors: non-invasive blood pressure cuff (NIBP), fetal heart rate monitor, and pulse oximeter connected. One peripheral IV connected between the mannequin and IV fluid.

Part 2 – Mannequin as above on operating room table. Awake, breathing spontaneously with standard monitors attached. Peripheral IV in situ connected to IV fluid. Operating room equipment (anesthesia machine, tables, equipment trays, boom lights) uncovered and available.

Please refer to Appendix A for a detailed list of all required equipment.

Personnel

Simulation instructor

Simulation technician

Confederate nurse – acting as scrub nurse in operating room

Confederate partner (optional)

Confederate respiratory therapist or anesthesia assistant (optional)

Learners

Senior anesthesiology resident – attending anesthesiologist

Senior OB/GYN resident – attending obstetrician

Mid-level OB/GYN resident – obstetric resident

Obstetric nurse – obstetric nurse

The baseline state of the mannequin is described in Table 2.

**Table 2:** Initial Parameters BPM: beats per minute; BID: twice per day; GBS: group B Streptococcus; GU: genitourinary; HEENT: head, eyes, ears, nose, and throat; HPI: history of present illness; HR: heart rate; OB: obstetrics.

Initial Presentation			
Initial vital signs	Blood Pressure: 130/80; Maternal heart rate: 105 BPM; Respiratory rate: 16; Temperature: 37^o^C; Fetal heart rate: Electronic fetal monitor showing HR of 130 BPM with no accelerations followed by a sudden decline to 100 BPM and persisting.		
Overall appearance	Obstetrical mannequin will be in semi-reclined labour bed. The patient is awake and alert. She is not distressed but becomes concerned of the status of the fetal heart rate tracing when the nurse recognizes the change or if the alarm sounds.		
Actors and roles in the room at case start	Patient: High fidelity mannequin, being voiced from control room with pregnant abdomen; Partner/support person: Confederate actor (optional); Nurse: Nurse		
HPI	Nurse is privately pre-briefed prior to scenario start (see Learner Preparation). The patient and partner (optional confederate) are aware of her medical history (see below). If asked they will state that she is 33 years-old and has had a previous vaginal delivery. Previously, she had an epidural and this time would like to try to have a more natural birth. She “broke her water” at home about three hours ago and has been having contractions since.		
Past Medical/Surgical History	Medications	Allergies	Family History
OB – This pregnancy has been uncomplicated. She developed gastroesophageal reflux at 36 weeks. Swab confirmed GBS-positive status. No history of hypertension or diabetes. Previous spontaneous vaginal delivery at term. She elected for epidural analgesia at 5 cm due to significant discomfort with contractions. She has laboured for 10 hours and pushed for 90 minutes.	Ranitidine, 150 mg BID; Prenatal vitamins	None	None, including no history of problems with anesthetics
Physical Examination			
General	Alert, oriented, conversational, concerned about fetal heart rate		
HEENT	Normal		
Neck	Normal		
Lungs	Breath sounds equal bilaterally and clear to auscultation		
Cardiovascular	Normal heart sounds, equal pulses bilaterally		
Abdomen	Pregnant		
Neurological	Normal		
Skin	Normal		
GU	Cervix is 5 cm dilated. The fetal orientation is vertex. There is a cord presentation.		
Psychiatric	Alert, concerned, anxious		

Ideal scenario flow

The nurse participant starts in the simulated labour room with the patient (mannequin) and her partner (optional confederate). The patient/partner will initiate conversation with the nurse to make the simulation seem more ‘natural’ or familiar. The fetal heart rate tracing will display a baseline of 130 BPM without accelerations or decelerations. The tocodynamometer will show contractions every three minutes. During the next contraction, the patient will moan and the fetal heart rate will decrease to 100 BPM. This will persist after the contraction is over. The patient will recover from the contraction. The nurse should identify the tracing abnormality and communicate the concern to the patient and initiate usual maneuvers to reposition the patient and apply supplemental oxygen. The fetal heart rate will remain low despite efforts. The nurse should call for help. The OB/GYN resident will respond. The nurse should brief the resident on the situation and their concerns. The resident should obtain verbal consent to perform a vaginal examination and do so during which he/she should identify a prolapsed umbilical cord and apply pressure to the fetal head to minimize cord compression (the fetal heart rate will maintain at 100 BPM). The diagnosis and treatment (emergency cesarean section) will be communicated to the patient and team. Consent will be obtained from the patient/partner. Pressure on the fetal head to prevent cord compression should be maintained for the remainder of the scenario. The importance of this maneuver should be communicated to the patient. Upon calling the control room to initiate an emergency cesarean section, control room personnel will enter and tell the nurse to prepare to assist the anesthesiologist and that they will ‘move’ the patient to the operating room. The mannequin will be moved or the room will be converted into an operating room. Remaining team members (Anesthesiology and OB/GYN attendings) will arrive at the operating room. The fetal heart rate will now be 80 BPM. The team will perform introductions and summarize the case. The anesthesiologist should perform a directed history and physical examination in preparation for a general anesthetic. The team should perform an abridged surgical safety checklist and request pediatrics to be paged. A general anesthetic will be administered, followed by endotracheal intubation. A cesarean section will ensue and the scenario will end with delivery. Table 3 provides notes for the instructor based on branching points during the scenario based on participant actions.

**Table 3:** Flow of the Scenario BPM: beats per minute; NICU: neonatal intensive care unit; OB/GYN: obstetrics/gynecology.

Instructor Notes – Changes and Case Branch Points		
Intervention / Time point	Change in Case	Additional Information
If the nurse performs a vaginal examination and identifies cord prolapse		Help will only come if she asks the partner to leave the room and find help or if she stops applying pressure to the fetal head and calls for help.
If no vaginal examination is performed	Change fetal heart rate to 80 BPM	The patient and/or partner should become increasingly anxious and vocal about the well-being of the fetus.
Any application of pressure to the fetal head	Change fetal heart rate to 100 BPM	
Stopping pressure on fetal head	Change fetal heart rate to 60 BPM	
Emergency cesarean section is called in to control room	Control room personnel will: Notify anesthesiology and OB/GYN attendings Initiate setting change from labour room to operating room. Ask nurse to go step out of the room and that her next role will be to assist the anesthesiologist	Refer to section “Setup: Equipment/Environment”
In operating room	Change fetal heart rate to 80 BPM	Adding a sense of urgency after a room change should improve engagement through emotional realism
If at any point verbal consent is not received: Vaginal examination Prolonged vaginal examination to maintain pressure on fetal head Cesarean section General anesthetic	The patient or partner should respond outraged that they are not being involved	
Cesarean section delivery without paging pediatrics or NICU team	Scrub nurse (confederate) will state there is no one here to receive the baby End scenario	
Cesarean section delivery with pediatrics or NICU team	End scenario	

Critical actions

The nurse recognizes an abnormal fetal heart rate.

The nurse should attempt to remedy the reduced fetal heart rate with supplemental oxygen and positional change.

When clear that fetal heart rate pattern is recalcitrant to initial interventions, the nurse will call the OB/GYN resident to assess the patient.

The OB/GYN resident should request/initiate the above maneuvers if not done so by the nurse and perform a vaginal examination to produce a diagnosis.

The nurse should advocate for a vaginal examination if not initiated by OB/GYN resident. 

The nurse should also advocate for attending OB/GYN to come if the resident is unable to resuscitate on his/her own or develop an adequate plan.

The OB/GYN resident should communicate the diagnosis to the nurse and request attending OB/GYN staff and anesthesiologist and preparation for an emergency cesarean section.

The OB/GYN resident should communicate the diagnosis to the patient, the rationale for prolonged vaginal examination, and need for delivery by cesarean section.

Once the team is available, there should be a transference of leadership, a handover, and a clear plan of action. 

The team should engage in a succinct surgical safety checklist prior to cesarean section. 

The anesthesiologist should perform a directed history and physical examination in preparation for general anesthetic.

If the anesthesiologist offers a spinal or epidural, the OB/GYN should advocate that the cesarean section should start immediately and request the fastest method of anesthesia.

The anesthesiologist will induce the patient using a general anesthetic and perform endotracheal intubation.

The nurse will assist the anesthesiologist in the induction of general anesthesia.

Anticipated management errors

The following is a list of management errors or difficulties that are commonly encountered when using this simulation case.

Failure To Recognize Fetal Heart Rate Changes

At any point that there is a significant delay in recognizing fetal heart rate changes, the partner (confederate) will verbalize that the fetal heart rate tracing has changed.

Failure To Recognize Cord Presentation

If this situation presents wherein either the nurse or resident do not perform a vaginal examination or an examination fails to identify the prolapsing cord, the fetal heart rate will fall and maintain at 80 BPM. This will prompt requesting the attending OB/GYN for assessment or proceeding with an emergency cesarean section. Discussing the differential diagnosis of intrapartum fetal heart rate changes during the summary phase of debriefing may foster discussion on the topic.

If the resident requests application of a fetal scalp monitor, one will be provided. The resident will be allowed to apply it and there will be no change to the fetal heart rate.

Failure To Call For An Emergency Cesarean Section

The fetal heart rate will continue to worsen (as low as 60 BPM) until a code is called.

Cessation Of Pressure On Fetal Head

The fetal heart rate will reduce to 60 BPM and continue to worsen (by 30 BPM every 30 seconds).

If the pressure is reapplied or the resident requests the nurse to exchange positions with them and the nurse maintains pressure on the fetal head, the fetal heart rate will recover to 100 BPM (in labour room) or 80 BPM (in the operating room).

Assessment and debriefing guide

Assessment

This scenario was developed as a formative assessment tool with a focus on nontechnical skills and crisis resource management. There are multiple suitable assessment methods that can be used. We utilize a performance checklist, as well as a Crisis Resource Management Checklist (Ottawa GRS) [3]. Please refer to Appendix B for the performance checklist.

Debriefing

We suggest an interprofessional, multidisciplinary team debriefing guided by an experienced simulation instructor. The goal of the debriefing is to allow learners to actively reflect on their own and the team’s performance, which is an essential step in adult experiential learning. Instructors should strive to create a safe, supportive, and respectful environment where all learners are encouraged to participate. Debriefing should focus on the educational objectives, both technical and non-technical. We advocate for the use of the promoting excellence and reflective learning in simulation (PEARLS) framework to organize the debriefing [5]. Where available, we advocate for the use of video review during the debriefing. Be cognizant of timing; the debriefing should take 30-40 minutes. Strategies to debrief common errors can be found in Table 4, and a complete debriefing guide can be found in Appendix C.

**Table 4:** Common Errors and Debriefing Strategies OR: operating room; SBAR: Situation-Background-Assessment-Recommendation.

Error Type	Common Errors Observed	Solutions (Teaching Points)
Technical Skills (Medical Knowledge, Clinical Skills)	Does not include umbilical cord prolapse in the differential diagnosis	Directive feedback or group collaborative effort to develop a differential diagnosis for intrapartum fetal heart rate changes.
	Delay in surgical delivery	Allowing learner to explore and reflect on the ramifications of delay. This is an excellent opportunity to use the advocacy inquiry technique.
	Does not provide continued pressure on the fetal head to participate in other activities	Directive feedback with a description of the pathophysiology of fetal heart rate abnormality during umbilical cord prolapse.
	Failure to perform surgical safety checklist	Directive feedback and review of an abridged version for use in emergency situations.
Non-Technical Skills (Crisis Resource Management)	Poor communication of the diagnosis to the patient.	Receiving feedback from the voice actor Advocacy inquiry style exploration or role-playing example.
	Ignoring partner	Allowing the learner to visualize themselves in a partner role to explore those experiences and expectations.
	Poor communication between team members	Facilitating team discussion; use of video examples. Description of various handover tools (ie, SBAR). Exemplifying ‘closed-loop communication’. Discuss sharing one’s mental model or cognitive frame with specific examples.
	Failure to recognize poor fetal heart rate tracing	Discuss situational awareness – for example, scanning of monitors at critical moments, before/after transfer to the OR, before/after any significant procedure, during any acute change in vital signs.
	Failure to manage human resources	Discuss each member’s human resource wants during the scenario and brainstorming hypothetical redistributions.

Program evaluation

We emphasize collecting evaluative data from participants after their simulation sessions. Our evaluation tool can be found in Appendix D. The results of our initial program evaluation can be found in Table 5, where the numbers in each column represent the number of participants who chose each number on the Likert scale. As program evaluation, this survey was exempt from IRB approval.

**Table 5:** Evaluation Data (n = 19 Participants) CanMEDS: Canadian Medical Education Directives for Specialists

	1 (strongly disagree)	2	3	4	5 (strongly agree)
The objectives were made clear			2	3	14
The scenarios were relevant to my practice					19
The simulation team behaved in an appropriate and believable manner during the scenario			1	2	16
There was sufficient time allotted for hands-on participation and group interaction				4	15
The staff met the stated learning objectives				3	16
The staff were knowledgeable and informed				4	15
The staff provided adequate and appropriate feedback				2	17
The debriefing sessions were logically organized and clarified important issues				2	17
The knowledge gained from this session will enhance/influence my practice				1	18
The session helped increase my confidence in treating patients when a crisis occurs			1	2	16
I would like to attend additional simulation sessions				2	17
Of the 19 participants, the following identified these standard Canadian Anesthesia Society 2005 monitoring roles as having been addressed during the session					
Medical Expert			18		
Collaborator			15		
Professional			6		
Health Advocate			2		
Communicator			19		
Manager			5		
Scholar			0		
Please take a moment to reflect on your previous experience in both simulation and clinical practice. Do you think that simulation has helped your clinical practice?					
Yes			19		
No			0		

## Discussion

This scenario, as well as the rest of the ‘OB/GYN Anesthesia Nursing Simulation Curriculum’ (Table 1), was developed to allow learners to practice managing rare and important clinical scenarios that they may not experience sufficiently during their period of training to achieve competence. The perinatal morbidity and mortality of improperly managed intrapartum umbilical cord prolapse are significant. This crisis requires immediate intervention and limits the healthcare team by occupying one member to apply constant pressure to the fetal head. Furthermore, this team member is often the team leader and must effectively transfer leadership and assume a very different role. This scenario provides an excellent clinical context to hone non-medical acumen.

This scenario has undergone several iterative changes during practice and implementation. Foremost was reducing the number of simulation rooms to one. Several logistical challenges were associated with a two-room setup: scheduling adjacent theatres, relocating control room staff, and transferring of the mannequin (between rooms and to an operating table). This setup necessitates a wireless mannequin, two control rooms, and transference of control room staff without interrupting or interfering with the scenario. Use of a single room in which the operating theatre equipment was concealed and the lighting could be adjusted was effective and practical. When participants went to scrub, the “labour room” could be quickly converted into an “operating room”. Additionally, we identified key discussions that took place while scrubbing which were often referenced during the debriefing. Overall, participants felt this scenario would improve their clinical practice.

There are limitations to this modality of teaching. Simulation requires many resources, including equipment, instructor and learner time, and instructor expertise. It is also heavily dependent on learner engagement and their willingness to participate and share their experiences with their colleagues. With the appropriate support from departments and learners, along with adequate preparation, we feel that it is an invaluable resource, especially for teaching around rare medical conditions and crisis resource management skills.

## Conclusions

We describe the design and implementation of an interprofessional and multidisciplinary simulation scenario to teach OB/GYN residents, anesthesiology residents, and nurses about the management of intrapartum umbilical cord prolapse. Debriefing points and common pitfalls are elaborated along with program evaluation data. This scenario is one component of a comprehensive theatre-based simulation curriculum, the goal of which is to provide formative assessment around less common obstetrical emergencies, and crisis resource management.

## Appendices

Appendix A: Equipment

General

Two simulation rooms (#1 set up as a labour room, #2 set up as an operating room (OR))

or

One simulation room (initially set up as a labour room with hidden OR equipment in the periphery that will be uncovered and moved into place while the team moves to the OR)

Mannequin

- SimMom® (Laerdal Medical Corp., Wappingers Falls, NY, USA) (wig, pregnant abdomen with baby inside) on stretcher, awake, and breathing spontaneously

- 1 x 18 G intravenous in situ connected to 1-liter crystalloid

- Patient chart with antenatal records.

Monitors (initial)

- Non-invasive blood pressure cuff (NIBP) cycling every three minutes

- Pulse oximeter

- Electrocardiogram

- Fetal heart rate (FHR) monitor

- Tocodynamometer

Operating Room

- Operating table

- NIBP cycling every three minutes

- Pulse oximeter

- Electrocardiogram

- FHR monitor

- Arterial line (available, not attached to mannequin)

Anesthesia Equipment (in OR)

- Ventilator and anesthetic machine turned off

- Standard monitors as above

- Breathing circuit and mask

- Suction and Yankauer

- Gas supply for air, O2

- Anesthetic agent (sevoflurane or desflurane)

Airway equipment:

- Laryngoscope

- Oral airway

- Endotracheal tubes (ETT) #6, #7

- Stylet

- Laryngeal mask airways (LMA) #4, #5

Breathing:

- Stethoscope

Circulation:

- 1-liter bags of crystalloid x 2

- IV infusion line x 2

- Blood set x 1

Induction drugs:

- Fentanyl or sufentanil

- Propofol

- Ketamine

- Succinylcholine, rocuronium

Various size syringes

Spinal Setup

Epidural Set Up

Surgical Equipment (in OR)

- Copy of surgical safety checklist

- Cesarean section tray (NOTE: a basic operating tray should be present to adequately simulate an obstetrical operating room. However, the actual steps of cesarean section are not part of the objectives for this scenario.)

- Wedge

- Laparotomy drape + clips x2 (to hang)

- Prep solution

- Foley catheter, bag

- Lap-pads/Sponges

- #10 Blade on handle

- 2x Kocher clamps

- Doyen’s retractor/lower-end retractor/large abdominal wall retractor

- 4x Kelly clamps

- 1x Umbilical cord clamp

- 2x Green-Armytage clamps

- Needle driver

- Suture (does not need to be opened)

Personal protective equipment:

- Gowns x4

- Gloves x4 pairs

- Caps/Bouffant

Appendix B: Performance checklist

Anesthesiology

Performs a focused history and physical examination eliciting relevant information regarding:

- Presenting problem

- Personal health history

- Maternal and fetal vital signs

- Assessment of head and neck in preparation for intubation

- Initiates resuscitation of mother and fetus

- Ensures IV access, standard Canadian Anesthesia Society (CAS) monitoring, supplemental oxygen, and FHR monitoring

- Ensures the patient is appropriately positioned to minimize aortocaval compression

Conducts safe induction of general anesthesia for emergent cesarean section:

- Calls for help

- Prepares for potentially difficult airway with appropriate positioning and ensuring airway adjuncts available

- Ensures entire team is prepared for immediate cesarean section prior to induction of anesthesia

Obstetrics

Performs a focused history eliciting relevant information regarding:

- Presenting problem

- Current pregnancy

- Past medical history

- Current interventions to remedy abnormal fetal heart rate pattern

Performs a focused physical examination eliciting relevant information regarding:

- Fetal heart rate tracing

- Cervical examination

- Recognizes umbilical cord prolapse

- Communicates diagnosis

- Requests attending OB/GYN

- Requests anesthesiologist

- Requests emergency cesarean section

- Communicates clearly with patient regarding diagnosis, importance of pressure against fetal head, and emergency cesarean section

- Transfers leadership clearly

Nurse

Recognizes abnormal fetal heart rate pattern

Initiates resuscitation of fetus:

- Changes patient position

- Provides supplemental oxygen

- Fluid resuscitation

Call for OB/GYN assessment

Requests attending OB/GYN if no clear plan established by OB/GYN resident

Communicates diagnosis and plan of care to patient/support person if resident does not do so

Reassures and emotionally supports patient

Appendix C: Debriefing Guide

The following is adapted from the Promoting Excellence and Reflective Learning in Simulation (PEARLS) Debriefing Guide [5]

Pre-briefing

Prior to the session, the instructor should conduct an orientation session covering the following:

- Confidentiality: Instructors should sign a confidentiality form agreeing not to discuss the learners’ performance during the scenario or discussion during the debriefing outside of the simulation environment. Learners should sign a confidentiality form agreeing not to discuss any aspects of the case or debriefing outside of the simulation environment.

- Equipment: Instructors should describe the function of the simulation mannequin (what types of procedures can be performed, where to feel for pulses, where to listen for breath sounds, etc.), how to operate the monitors in the room, and how to get extra resources (location of phone, phone number to call, resuscitation cart, difficult airway cart, etc.).

- Fidelity: The learners should be encouraged to treat the scenario as they would a real clinical experience. Learners should be advised that if they are unsure whether a clinical finding or event is intended or an artifact to state this aloud so they can be appropriately directed.

Debriefing

Reactions phase: allows learners to express their initial thoughts and feelings

Tips:

- Ensure all learners are able to express themselves

- Make note of any positive or negative emotions, major conflicts, or tensions that can serve as a point of discussion during the ‘Analysis’ phase

- Ask open-ended questions: “How are you feeling?”

Descriptive phase: ensures all participants understand the major medical issues and key events of the case

Tips:

- Ask one or two participants to summarize the case in two to three sentences to avoid a detailed play-by-play of the scenario

- Ensure participants from all disciplines are able to contribute

- Make note of any disagreements between the learners as to the medical content (differential diagnosis, management plan, etc.) that can serve as a point of discussion during the ‘Analysis’ phase

- Clarify any misconceptions, correct any errors regarding the main presentation and diagnosis

Analysis phase: allows learners to practice reflective learning by exploring and analyzing positive performances and performance gaps

Tips:

- Be genuinely curious!

- Maintain the basic assumption that everyone is smart and is doing their best for the patient!

- Focus on two or three points, including both technical and non-technical skills, in your discussion

- Include discussion points based on your own observation as well as those generated by the learners in the ‘Reactions’ and “Descriptive’ phases

- Select appropriate tools, including advocacy-inquiry, plus/delta, and directive feedback, according to the type of performance gaps observed, the time available, the experience and insight of the learners, and your experience as a debriefer.

“What went well? What would you have wanted to change or do differently?”

“What are some pros and cons of…. [observed action or behaviour]?”

“What was going through your mind when you did/said…?.”

“What was your differential diagnosis?”

Normalize mistakes

“We specifically put you into a challenging situation and we did not expect you to manage everything perfectly”

“In our normal practice, we are used to those around us acting in a specific way, things are different in the simulator”

Generalize to clinical practice

“Have you ever experienced anything similar in your practice?”

“What strategies have you seen people use in your clinical experience?”

Before moving to the ‘Summary’ phase ask the learners if there are any other issues they would like to discuss

Summary phase: allows learners to review and summarize what was learned and to apply this to their clinical practice and allows instructors to confirm if the learning objectives of the scenario were achieved

Tips:

- Avoid bringing up new discussion points or topics

- Ask the learners to summarize their main take home points, i.e. “Tell me one thing you did well, one thing you would do differently, and one thing you learned that you will apply to your clinical practice”

- Summarize the main points of the discussion

- Ensure everyone contributes, even learners who participated as confederates can share what they learned from participating in the scenario and debriefing

Appendix D: Evaluation form

Multidisciplinary Simulation

Date: _____________________________  Discipline: ____________________________        

Evaluation of the Session:

1 = strongly disagree, 2 = disagree, 3 = neutral, 4 = agree and 5 = strongly agree

1) The objectives of the session were explicitly stated                             1           2           3            4           5           N/A

2) The objectives were met by the simulation staff                                   1           2           3            4           5           N/A

3) Staff were knowledgeable and informed                                               1           2           3            4           5           N/A

4) There was sufficient time dedicated to hands-on                                 1           2           3            4           5           N/A

     practice

5) During the debriefing, the staff provided appropriate                          1           2           3            4           5           N/A

     and timely feedback

6) The debriefing session was organized and helped                              1           2           3            4           5           N/A

     clarify important issues with the case

7) The knowledge gained from this session will influence                       1           2           3            4           5           N/A

     my practice

8) I have gained increased confidence in my capacities                          1           2           3            4           5           N/A

     to provide care to patients in acute emergencies

9) I would enjoy having more opportunities for practice                          1           2           3            4           5           N/A

Evaluation of the Debriefers:

1 = strongly disagree, 2 = disagree, 3 = neutral, 4 = agree and 5 = strongly agree

Name of Debriefers: ________________________________________                                                 

1) The debriefers provided a safe environment to share and              1           2           3            4           5           N/A

     discuss

2) The debriefers demonstrated professionalism throughout             1           2           3            4           5           N/A

      the debriefing

3) The debriefers were engaging and facilitated my learning             1           2           3            4           5           N/A

Evaluation of the Scenario:

1 = strongly disagree, 2 = disagree, 3 = neutral, 4 = agree and 5 = strongly agree

1) The scenario was relevant to my practice                                                  1           2           3            4           5           N/A

2) The clinical encounter had high realism                                                    1           2           3            4           5           N/A

3) The degree of difficulty of the scenario was appropriate                        1           2           3            4           5           N/A

      for my level of expertise

4) The case construct was conceptually sound                                            1           2           3            4           5           N/A

      (i.e., it made sense…)

If you have answered ≤ 2 to any of the previous statements, please briefly justify:                                                                                              

Global Evaluation of the experience:

1 = strongly disagree, 2 = disagree, 3 = neutral, 4 = agree and 5 = strongly agree

1) This was an effective use of my time                                                     1           2           3            4           5           N/A

2) I would appreciate having more simulation sessions                          1           2           3            4           5           N/A

3) I have gained knowledge through this experience                             1           2           3            4           5           N/A

4) What was the most useful or important lesson you learned during this session?

5) Can you identify any component of this session that would need to be improved in order to make the learning experience even better?

6) Please indicate which CanMeds roles were addressed during today’s session (check all that apply)

☐ Medical Expert

☐ Collaborator

☐ Professional

☐ Health Advocate

☐ Communicator

☐ Manager

☐ Scholar
